# Controlled Synthesis and Enhanced Gas Sensing Performance of Zinc-Doped Indium Oxide Nanowires

**DOI:** 10.3390/nano13071170

**Published:** 2023-03-25

**Authors:** Che-Wen Yu, Hsuan-Wei Fu, Shu-Meng Yang, Yu-Shan Lin, Kuo-Chang Lu

**Affiliations:** 1 Department of Materials Science and Engineering, National Cheng Kung University, Tainan 701, Taiwan; n56091352@gs.ncku.edu.tw (C.-W.Y.); n56091556@gs.ncku.edu.tw (H.-W.F.); n56074287@gs.ncku.edu.tw (S.-M.Y.); n56114053@gs.ncku.edu.tw (Y.-S.L.); 2 Core Facility Center, National Cheng Kung University, Tainan 701, Taiwan

**Keywords:** indium oxide, nanowires, chemical vapor deposition, doping, resistivity, gas sensing

## Abstract

Indium oxide (In_2_O_3_) is a widely used n-type semiconductor for detection of pollutant gases; however, its gas selectivity and sensitivity have been suboptimal in previous studies. In this work, zinc-doped indium oxide nanowires with appropriate morphologies and high crystallinity were synthesized using chemical vapor deposition (CVD). An accurate method for electrical measurement was attained using a single nanowire microdevice, showing that electrical resistivity increased after doping with zinc. This is attributed to the lower valence of the dopant, which acts as an acceptor, leading to the decrease in electrical conductivity. X-ray photoelectron spectroscopy (XPS) analysis confirms the increased oxygen vacancies due to doping a suitable number of atoms, which altered oxygen adsorption on the nanowires and contributed to improved gas sensing performance. The sensing performance was evaluated using reducing gases, including carbon monoxide, acetone, and ethanol. Overall, the response of the doped nanowires was found to be higher than that of undoped nanowires at a low concentration (5 ppm) and low operating temperatures. At 300 °C, the gas sensing response of zinc-doped In_2_O_3_ nanowires was 13 times higher than that of undoped In_2_O_3_ nanowires. The study concludes that higher zinc doping concentration in In_2_O_3_ nanowires improves gas sensing properties by increasing oxygen vacancies after doping and enhancing gas molecule adsorption. With better response to reducing gases, zinc-doped In_2_O_3_ nanowires will be applicable in environmental detection and life science.

## 1. Introduction

Gas sensing devices play a crucial role in detecting gases that are harmful to the environment or human health. Some of these gases are present at low concentrations and are colorless and odorless, making them difficult to detect. This highlights the significance of effective gas sensing devices. Indium oxide (In_2_O_3_) has been explored for properties such as its wide energy gap [1], low resistivity, and good catalytic activity. Bulk-scale indium oxide has several potential applications, including solar cells [2], organic diodes [3], metal reflective coatings [4], nanoscale gas sensors [5], and artificial synapses [6].

Indium oxide is an intrinsic n-type semiconductor with numerous oxygen vacancies [7,8], increasing the adsorption of oxygen and forming superoxide radicals (O_2_^−^, O^−^, O^2−^) on the surface [9]. The surface energy band bends upward, forming a depletion layer and narrowing the electron transport channel, making it suitable for semiconductor gas sensors [10,11]. When an n-type semiconductor reacts with a reducing gas, the gas sends electrons to the surface of the material, which narrows the electron depletion layer, widens the electron channel, and decreases resistance. Conversely, when it reacts with an oxidizing gas, the electron depletion layer widens and resistance increases.

There are many methods for synthesizing indium oxide nanowires, such as pulse laser deposition [12], the electrospinning method [13], the template method [14], and chemical vapor deposition [15,16,17]. In this study, chemical vapor deposition was used to synthesize the Zn-doped indium oxide nanowires, as it is an easily controlled method for forming the desired morphology. Here, we use zinc as a dopant, because the introduction of zinc increases the number of oxygen vacancies and is expected to improve the sensitivity and selectivity of the gas sensor. The atomic size of zinc is slightly smaller than that of indium [18]; thus, it can be used as a substitutional doping element. Single crystalline zinc-doped indium oxide nanowires (Zn-In_2_O_3_) were grown by chemical vapor deposition using indium oxide and zinc oxide as precursors. The morphologies, structures, electrical properties, and gas sensing capabilities of Zn–In_2_O_3_ nanowires were evaluated at different zinc concentrations. The results of this study support the development of more effective gas sensors.

## 2. Materials and Methods

### 2.1. Synthesis of Zn-Doped In_2_O_3_ Nanowires

Zn-doped indium oxide nanowires were synthesized using a three-zone tube furnace (Lindberg MPH, Riverside, MI, USA) via the vapor–liquid–solid (VLS) mechanism depicted in Figure S1. Each zone of the tube furnace could be independently set to different operating temperatures or held at any temperature below 1150 °C for a given duration. The Si substrate on which nanowires would be deposited was cleaned thoroughly with acetone, isopropanol, and deionized water (DI water) using an ultrasonic oscillator. Subsequently, a 10 nm gold layer was deposited on the substrate as a catalyst using an e-beam evaporation system (ULVAC VT1-10CE). In the first heating zone, 0.02 g of zinc oxide and 0.02 g of carbon powder were added, while 0.08 g of indium oxide and 0.08 g of carbon powder were added in the third heating zone. The carbon powder acted as a reducing agent to reduce the zinc oxide and indium oxide powder to zinc and indium ions during the reaction. Prior to the experiment, argon gas at 500 sccm was introduced to the vacuumed furnace for 10 min to make the environment clean enough to grow nanowires. Afterwards, we annealed the furnace at the target temperature for 30 min, during which the argon and oxygen were introduced. The alterations of the substrate positions resulted in successful growth of the Zn-doped indium oxide nanowires.

To avoid the effect of thermal convection in the experiment, the first, second and third zones were heated to 950 °C, 925 °C, and 900 °C, respectively, within 35 min. After the heating process, Ar and O_2_ were introduced into the CVD system and system pressure was maintained at 1.9 torr for 30 min. Indium oxide nanowires doped with 3 at% Zn and 5 at% Zn were obtained after cooling the furnace to room temperature. Structural characterizations for the In_2_O_3_ and Zinc-doped In_2_O_3_ nanowires were conducted using AFE-SEM (Zeiss Auriga, CarlZeiss, Jena, Germany), HR-TEM (JOEL JEM-2100F CS STEM, Tokyo, Japan), EDS (Bruker, Billerica, MA, USA), and XPS (ULVAC-PHI 5000 Versaprobe, Chigasaki, Kanagawa, Japan).

In the XPS analysis, monochromatic Al Kα radiation was used, and the equipment was operated at 24.9 W with an energy resolution of approximately 0.5 eV. The takeoff angle was set at 45°. Survey spectra were collected with a passing energy of 1 eV, covering a range from 1295 eV to −3 eV. High resolution spectra were collected within the following ranges: 1052 eV to 1016 eV (Zn 2p), 541 eV to 525 eV (O 1s), 460 eV to 440 eV (In 3d), and 297 eV to 279 eV (C 1s). A passing energy of 0.2 eV was used for each scan. XPSpeak41 software was used to analyze all XPS spectra, and the Shirley method was employed to compute the background of high-resolution spectra.

### 2.2. Electrical Measurements

**Figure 1:**
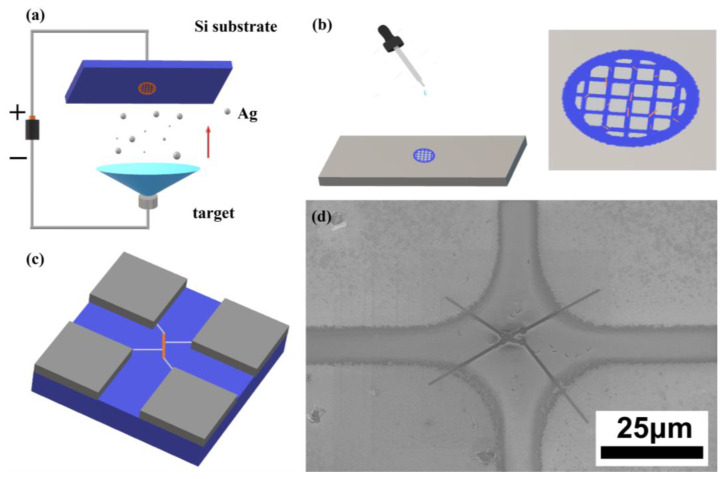
Schematic illustration for fabrication of the electrical measurements microdevice: (**a**) copper mesh was pasted on the cleaned substrate and a 200 nm layer of silver was deposited by e-beam evaporator; (**b**) nanowire solution was dripped onto the substrate with the pre-deposited Ag layer as a contact; (**c**) electrical measurement microdevice; (**d**) SEM image of the microdevice.

To measure the electrical resistivity of the nanowires, we utilized an e-beam evaporation system (ULVAC VT1-10CE) to deposit a 300 nm layer of silicon dioxide on the silicon substrates that had been cleaned ultrasonically, thus creating an insulation layer. We then applied a copper mesh as a mask and utilized the same e-beam evaporation system to deposit a 200 nm layer of silver, which served as a conduction layer (Figure 1a). Afterwards, we acquired the nanowire solution from the samples with an ultrasonic oscillator and dripped it onto the silver-coated substrate (Figure 1b). Finally, the nanowires were connected to the electrodes using a dual-beam focused ion beam system (FEI Helios G3CX) (Figure 1c,d). For accuracy, a measuring method proposed by Gu et al. [19] was utilized, the resistance of each section of the nanowire was measured, and the actual resistivity was calculated by subtracting the contact resistance.

### 2.3. Gas Sensing Measurements

Firstly, Si substrates were ultrasonically cleaned with acetone, isopropanol and DI water and then plated with a SiO_2_ layer using an e-beam evaporation system (ULVAC VT1-10CE). Afterwards, a copper wire was applied to the substrate as a shadow mask and a 200 nm silver layer was deposited using the same e-beam evaporation system (ULVAC VT1-10CE). Removal of the copper wire resulted in a non-conductive channel in which the nanowire solution could be dripped. The method for acquiring the nanowire solution was the same as for the electrical measurements. The nanowires were then dried and platinum electrodes were attached to both ends of the nanowires using the dual-beam focused ion beam system (FEI Helios G3CX). The gas sensor was connected to a multifunction power meter and placed on a heating table to regulate its working temperature. The test structure was then placed in a cavity, and the gas flow was controlled with a mass flow controller. The gas balance was maintained using a vacuum pump, and resistance change at a fixed voltage was measured to determine the response value. To ensure the accuracy of the experiment and the reliability of the gas sensing, each test was repeated three times.

## 3. Results

### 3.1. Synthesis and Characterization of In_2_O_3_- and Zn-Doped In_2_O_3_ Nanowires

**Figure 2:**
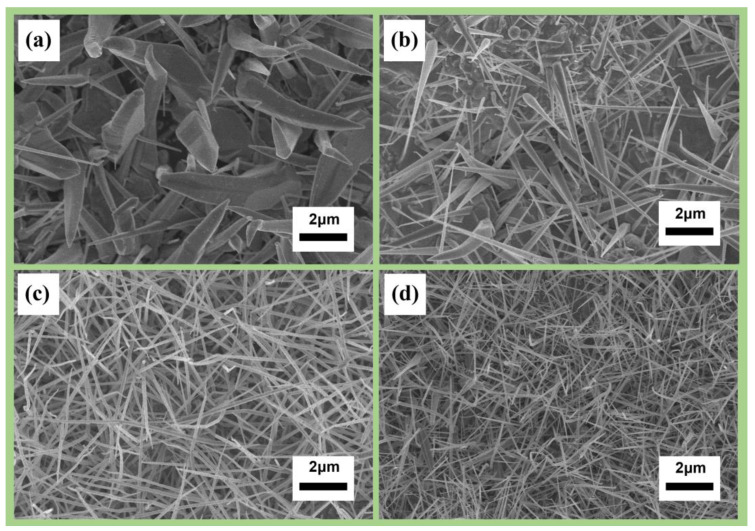
Morphologies of indium oxide nanowire synthesis at different temperatures and deposition positions: (**a**) 875 °C at 1 cm; (**b**) 810 °C at 3 cm; (**c**) 710 °C at 5 cm; (**d**) 650 °C at 7 cm.

A series of experiments were performed to investigate the influence of deposition position and temperature on the growth of nanowires. The experiments were conducted at four different deposition positions, 1 cm, 3 cm, 5 cm, and 7 cm from the three-zone tube furnace at deposition temperatures of 875 °C, 810 °C, 710 °C, and 650 °C, respectively. Figure 2 shows that the diameter of the nanowires was larger when the deposition temperature was 875 °C, resulting in a bamboo-like shape. The results indicate that high reaction temperatures lead to low nucleation free energy, a large nucleation radius, and thick nanowire growth, while lower reaction temperatures contributed to thinner nanowires. These findings demonstrate the crucial role deposition temperature plays in the growth of nanowires.

**Figure 3:**
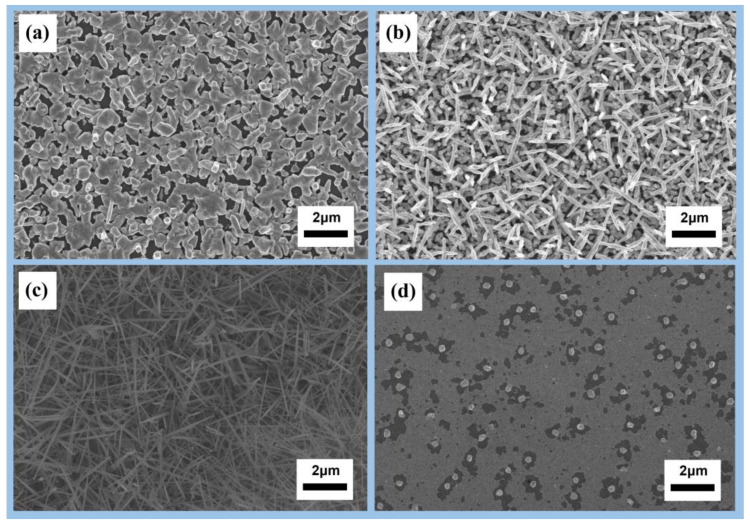
Morphologies of indium oxide nanowire synthesis at different gas flow rates: (**a**) 50 sccm Ar + 20 sccm O_2;_ (**b**) 70 sccm Ar + 20 sccm O_2;_ (**c**) 90 sccm Ar + 20 sccm O_2;_ (**d**) 110 sccm Ar + 20 sccm O_2_.

To explore the morphology of nanowires at different gas flow rates, the flow rates of argon were set at 50, 70, 90, and 110 sccm, while the flow rate of oxygen was kept constant at 20 sccm (Figure 3). With a low carrier-gas flow rate, the transport of the precursor was slow, leading to overdeposition of the precursor and the formation of coarse particles. Excess amounts of carrier gas resulted in a dilution of the precursor partial pressure, causing difficulties in depositing the precursors on the substrate. This indicates that a delicate balance must be maintained between the flow rates of precursor gases and carrier gas to achieve optimal nanowire growth.

**Figure 4:**
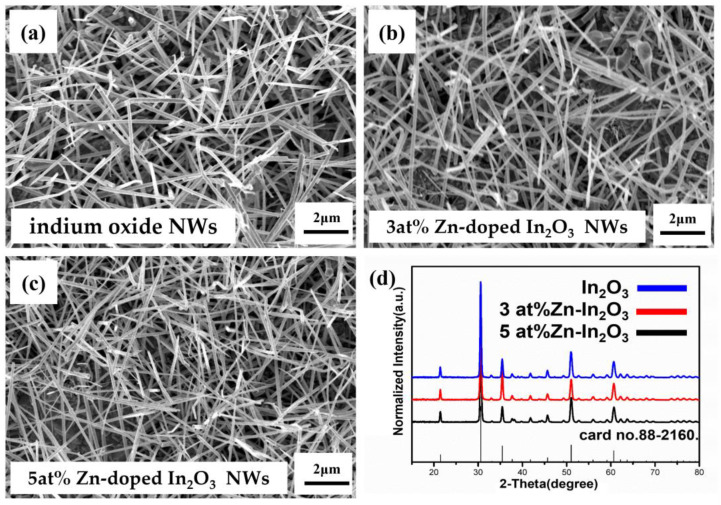
(**a**–**c**) SEM images of: (**a**) indium oxide nanowires; (**b**) 3 at% zinc-doped indium oxide nanowires; and (**c**) 5 at% zinc-doped indium oxide nanowires. (**d**) XRD analysis of In_2_O_3_, 3 at% Zn-In_2_O_3_, and 5 at% Zn-In_2_O_3_ nanowires.

To find the optimal conditions for Zn-In_2_O_3_ nanowire growth, we varied the deposition sites of ZnO and In_2_O_3_. The doping percentage at the lower stream was higher, while that at the upper stream was lower. Figure 4a–c shows scanning electron microscopy (SEM) images of undoped, 3 at% Zn, and 5 at% Zn nanowires, respectively. The SEM images in Figure S2 reveal zinc clusters above the gold catalyst, indicating a higher zinc concentration in the lower stream. The excess zinc supercooled and precipitated as clusters on gold particles of the In_2_O_3_ nanowires before the oxidation stage. The results show that the doping percentages were consistent with the expected values, demonstrating the effectiveness of the method.

**Figure 5:**
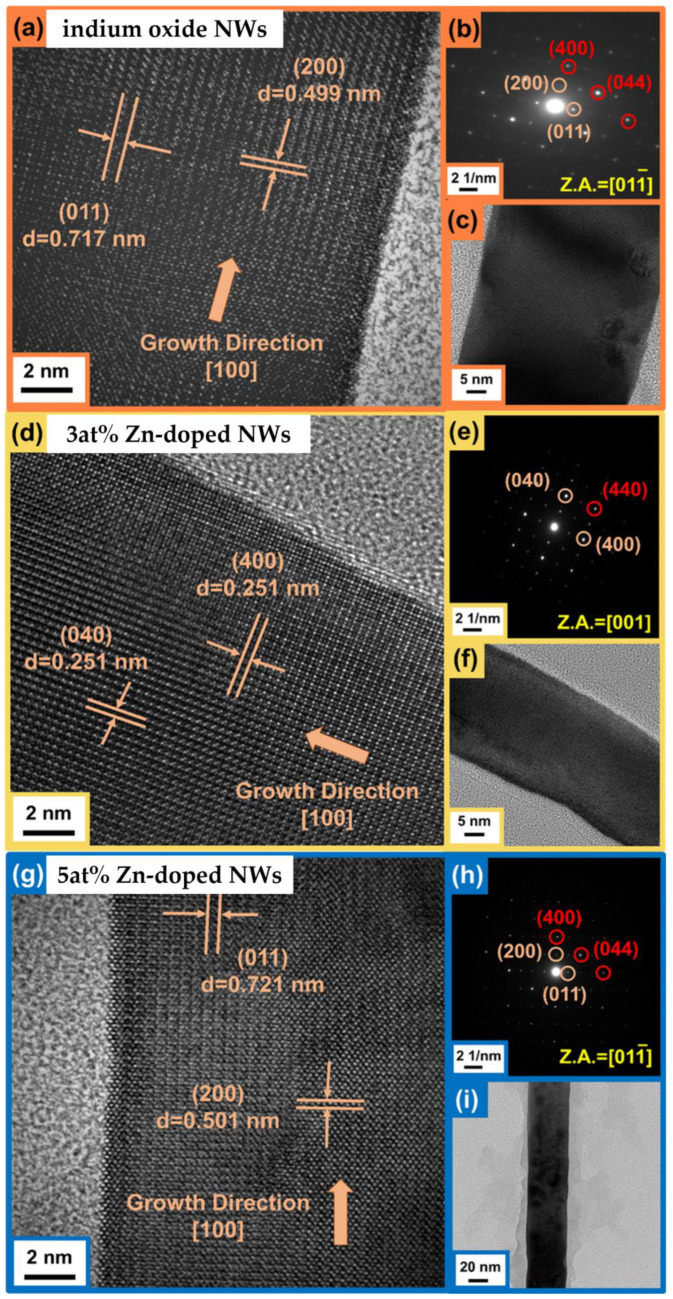
TEM analysis of single nanowire: (**a**) 800k HRTEM image; (**b**) SAED pattern; (**c**) 300k HRTEM image of In_2_O_3_ nanowire; (**d**) 800k HRTEM image; (**e**) SAED pattern; (**f**) 300k HRTEM image of 3 at% Zn-In_2_O_3_ nanowire; (**g**) 800k HRTEM image; (**h**) SAED pattern; (**i**) 300k HRTEM image of 5 at% Zn-In_2_O_3_ nanowire.

The microstructures of the In_2_O_3_ and Zn-In_2_O_3_ nanowires were characterized using high-resolution transmission electron microscopy (HRTEM). The HRTEM images (Figure 5) show that the crystal planes of In_2_O_3_ nanowires were (200) and (011), with interplanar spacings of 0.499 nm and 0.717 nm, respectively. The growth direction of the In_2_O_3_ nanowires was determined to be [100]. The HRTEM images of the 3 at% Zn- In_2_O_3_ nanowires show that the crystal planes were (400) and (040) with interplanar spacing of 0.251 nm and growth direction of [100]. The bright diffraction point in the selected area electron diffraction (SAED) pattern was (440). The HRTEM images of the 5 at% Zn- In_2_O_3_ nanowires show that the crystal planes were (200) and (011) with interplanar spacings of 0.501 nm and 0.721 nm, respectively. The growth direction was determined to be [100]. The bright diffraction points in the SAED pattern were (400), (222) and (044); indium oxide structures grown in this direction were observed. The composition and zinc distribution of the nanowires were analyzed using transmission electron microscopy and energy dispersive spectroscopy (TEM-EDS), with the results showing that the doping percentages were 2.9 at% and 4.9 at%, respectively, as shown in Figures S3 and S4.

X-Ray Diffraction (XRD) analysis was also performed, as shown in Figure 4d. The identified XRD peaks correspond to the expected face-centered cubic In_2_O_3_ structure, with peak positions at 30.62° (222), 35.52° (400), 45.78° (431), 51.13° (440), and 60.79° (622), confirmed with the JCPDS card number 88-2160. The bright diffraction points in the selected area electron diffraction pattern (SAED), such as those at (222), (400), and (044), correspond to the expected bixbyite phase of indium oxide, indicating that the structure of the nanowires did not change after doping with zinc.

**Figure 6:**
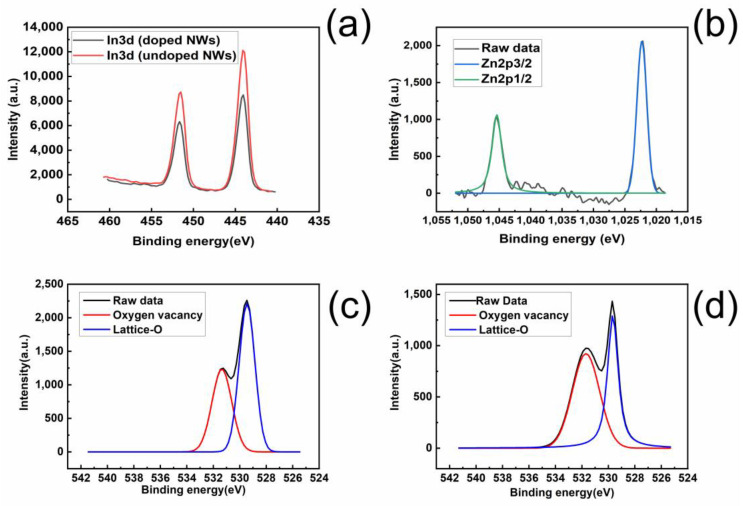
XPS analysis of substrate surface of: (**a**) undoped and zinc-doped In_2_O_3_ nanowire for In3d peaks; (**b**) zinc-doped In_2_O_3_ nanowire for Zn2p peaks; (**c**) undoped In_2_O_3_ nanowire for O1s peaks; (**d**) zinc-doped In_2_O_3_ nanowire for O1s peaks.

To study the composition of the nanowires, X-ray photoelectron spectroscopy (XPS) measurements were performed on the samples. The XPS spectra of In_2_O_3_ and Zn-In_2_O_3_ nanowires are shown in Figure 6. The In 3d curve of In_2_O_3_ shows two peaks at 444.0 ± 0.1 eV and 451.6 ± 0.1 eV, corresponding to In 3d_5/2_ and In 3d_3/2_ orbitals of trivalent indium ions (Figure 6a). The binding energy of oxygen to indium is approximately 529.6 ± 0.1 eV [20]. The XPS spectra of In_2_O_3_ and Zn- In_2_O_3_ (Figure 6c,d) show corresponding peaks at this intensity. There is an oxygen vacancy peak at 531.7 ± 0.1 eV, slightly lower than the oxygen lattice energy [15]. XPS is highly sensitive to changes in the oxidation state of the surface, and the area of the peak changes accordingly. The oxygen vacancy peak area of Zn-In_2_O_3_ is greater than that of In_2_O_3_ due to the increased oxygen vacancies in Zn-In_2_O_3_ caused by doping. The intensity of the lattice O of Zn-doped nanowires decreased compared with the undoped nanowires because the lattice mismatch between zinc atoms and indium atoms contributed to disordered arrangements of the lattice. In_2_O_3_ did not show any zinc-containing components or peaks in the analysis, as it was not doped with zinc. The binding energies of Zn-In_2_O_3_ in 2p_3/2_ and 2p_1/2_ orbitals are 1022 ± 0.1 eV and 1045.1 ± 0.1 eV, respectively, indicating the presence of zinc in the Zn-doped indium oxide nanowires (Figure 6b).

The electrical resistivity of the nanowires was measured using the method proposed by Gu et al. [19]. The technique was used to eliminate contact resistance and determine the actual resistivity of each section of the nanowire. The electrode configuration and resistance measurements of each segment of the nanowire are depicted in Figures S5–S7. It is important to ensure that the contact electrode and the probe make an ohmic contact and that the probe is thin enough.

Since indium oxide is an intrinsic n-type semiconductor, experiments must be performed in a dark chamber to prevent the light source from affecting the experimental resistance value. The light source can increase the conductivity of the materials by causing electrons to jump from the valence band to the conduction band after absorbing light energy. Table 1 shows that the resistivity of In_2_O_3_ nanowires was 1.07 × 10^−4^ Ω·cm, while the resistivity of 3 at% Zn- In_2_O_3_ nanowires was 2.67 × 10^−4^ Ω·cm and the resistivity of 5 at% Zn- In_2_O_3_ nanowires was 9.31 × 10^−4^ Ω·cm. The results indicate that the resistivity of the original nanowire was the lowest, and that the resistivity increased after doping. The zinc acted as an acceptor, which could increase the holes of the nanowire and offset the electrons of the n-type semiconductor indium oxide.

### 3.2. Gas Sensing Mechanisms

The number of adsorbed active sites on the surface of materials is critical for gas sensing applications. A larger number of active sites leads to an increase in the number of free electrons or holes, thereby enhancing the reaction of gas adsorbed on the surface. Adsorption can be classified into weak and strong chemical adsorption. Weak chemical adsorption refers to direct adsorption on the surface, which does not affect the electron hole concentration of the crystal. On the other hand, strong chemical adsorption involves the interaction of different gases with ions on the surface, contributing to the formation of different types of ions. The following experiments aimed to analyze the chemical reaction path of electron hole migration after exposing the nanowires to different gases.

When gas molecules meet the nanowires, they may either donate or remove charge carriers, leading to changes in the density of electrons or holes and in the conductivity of the nanowires. The doping ratio of metals in the nanowires was adjusted by varying the synthesis parameters, such as the type of precursor, deposition location, time, temperature, and gas flow rate. The change in the valence state of the metal generated oxygen vacancies on the surface. In this study, the gas sensing properties of indium trioxide (In_2_O_3_), 3 at% zinc-doped indium trioxide (3 at% Zn-In_2_O_3_), and 5 at% zinc-doped indium trioxide (5 at% Zn- In_2_O_3_) nanowires were investigated for the detection of carbon monoxide, acetone, and ethanol. The response of the nanowires to these gases and the reaction mechanism were analyzed and discussed.

In_2_O_3_ nanowires are n-type semiconductors, which exhibit changes in resistance when exposed to different gases. Upon exposure to reducing gases, the gas reacts with the adsorbed oxygen on the material surface, leading to a decrease in resistance due to the charge-transfer reactions between the gas and oxygen surface anions, which donate electrons to In_2_O_3_. Conversely, exposure to oxidizing gases results in an increase in resistance due to the charge-transfer reactions between gas and surface, which extract electrons from In_2_O_3_. We use the following formula to express the responsivity to the gas (Response, S):

(1) $$S=\left[1-\left(\frac{\mathrm{Rgas}}{\mathrm{Rair}}\right)\right]\times 100$$

where R_gas_ is the resistance value when gas is introduced and R_air_ is the resistance value when general air is introduced.

The reaction mechanism of the sensor was analyzed when exposed to acetone. Each acetone molecule reacts with different negatively charged oxygen ions, leading to the release of electrons. The more negatively charged oxygen ions that are produced on the surface, the greater the electron exchange will be. When the sensor is exposed to acetone, acetone molecules reacting with 8O^−^ will release 8e^−^, molecules reacting with 8O^2−^ will release 16e^−^, and molecules reacting with 4O_2_^−^ will release 4e^−^. The reaction between the acetone and the negatively charged oxygen ions is temperature-dependent, with the optimal temperature being around 250 °C, at which the negatively charged oxygen ion O^2−^ dominates the gas–solid interaction and is the main contributor to the reaction. It was also noted that enhanced acetone gas sensing performance can be understood from the important perspective of surface defects. The surface oxygen vacancies can act as preferential adsorption sites, resulting in more surface oxygen species being adsorbed on the nanowire surface. Hence, the presence of surface oxygen vacancies is essential to the acetone gas sensing performance of the nanowires.

The reaction of carbon monoxide (CO) with different negatively charged oxygen ions at different temperatures was also analyzed. At temperatures below 200 °C, CO may not have enough energy to overcome the activation energy, contributing to low responsivity without surface catalysis. At around 200 °C, the lower activation energy from O_2_^−^ dominates [21]. At high temperatures, the reaction is dominated by O^-^. Upon adsorption of CO gas molecules on the surface of the nanowires, they react with the negatively charged oxygen ions to form carbon dioxide (CO_2_).

In the case of ethanol, at low temperatures, ethanol molecules react with O_2_^−^ and O^−^ on the surface; O_2_^−^ is usually chemisorbed here. At elevated temperatures, both O^−^ and O^2−^ are usually chemisorbed, but O_2_^−^ disappears rapidly, leading to a good response. When ethanol gas molecules are adsorbed on the nanowire surface, they react with the negatively charged oxygen ions. The oxygen ions oxidize the ethanol, resulting in its decomposition into CO_2_ and H_2_O, which further increases the concentration of free electrons in the conduction band.

### 3.3. Gas Sensing Measurements

**Figure 7:**
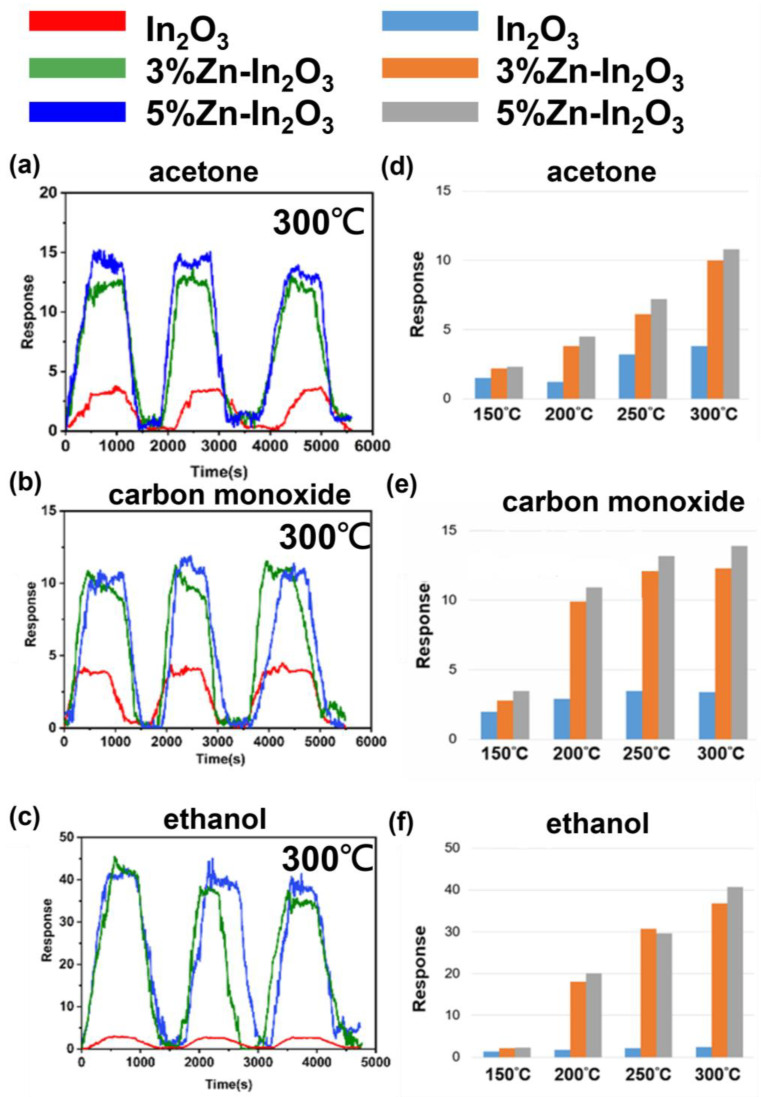
Dynamic responses at 300 °C of In_2_O_3_, 3 at% Zn-In_2_O_3_ and 5 at% Zn-In_2_O_3_ nanowires to 5 ppm of (**a**) acetone, (**b**) carbon monoxide, and (**c**) ethanol at optimal operating temperature, and bar graphs for different temperatures. Responses of In_2_O_3_, 3 at% Zn-In_2_O_3_ and 5 at% Zn-In_2_O_3_ nanowires to 5 ppm of (**d**) acetone, (**e**) carbon monoxide, and (**f**) ethanol at temperatures of 150–300 °C.

We investigated the gas sensing behavior of In_2_O_3_, 3 at% Zn- In_2_O_3_, and 5 at% Zn- In_2_O_3_ nanowires in response to 5 ppm of carbon monoxide, acetone, and ethanol at temperatures ranging from 150 °C to 300 °C. The dynamic response of the nanowires to the gases was evaluated using the resistance values shown in Figures S8–S10. After three measurements, the response remained almost the same, demonstrating the high reliability of the gas sensing. The average response of the nanowires to the gases is summarized in Table S1. The results reveal that the doping of In_2_O_3_ nanowires with Zn significantly improved their gas sensing performance, particularly to acetone gas. The nanowires doped with 5 at% Zn exhibited the best response, 3–4 times better than that of undoped indium oxide nanowires. The response to acetone gas increased with the temperature, with the best response being observed at 300 °C, as shown in Figure 7a–c.

The response of In_2_O_3_ nanowires to ethanol was found to be greatly enhanced after Zn doping, with responsivity increased to 10–13 times. A faster and more selective response of doped indium oxide nanowires to ethanol was observed compared with carbon monoxide and acetone. Additionally, the three nanowires had reversible reactions to the three gases. Tables S2 and S3 show the comparison of the results with previous studies on indium oxide nanomaterials using different dopants or structures [5,22,23,24,25,26].

### 3.4. Gas Sensing Enhancement

Factors related to the detection mechanism and the effective control of resistance values include the adsorption capacity of the surface, the number of active sites, electrical and chemical properties, thermodynamic stability, and catalytic activity. Here, the focus was on the principle of oxygen adsorption. At temperatures below 150 °C, oxygen in the atmosphere is adsorbed and reacts with n-type semiconductors, such as indium oxide nanowires, by obtaining electrons from their surface. In n-type semiconductors, this adsorption of oxygen ions leads to the formation of regions with fewer electrons near the surface, known as the electron depletion layer. This can be understood based on the electron core-shell structure, where the depletion region acts as the shell, and the channel through which electrons can flow in the central region is the core. The performance of reducing gases can be observed through a decrease in the surface oxygen ion concentration due to oxidation, which injects the extracted electrons back into the semiconductor and reduces the thickness of the electron depletion region, increasing the channel width and conductivity, as shown in Figure S11. On the other hand, when exposed to an oxidizing gas, the gas molecules remove electrons from the surface of the material, resulting in an increase in the electron depletion region and a decrease in the channel width, contributing to increasing resistance.

In this study, the effects of zinc doping on indium oxide nanowires for gas sensing are analyzed. Firstly, the introduction of zinc doping into indium oxide nanowires increases the number of active sites through the formation of defects, such as oxygen vacancies. This enhances the response of nanowires to various gases; at certain concentrations, the optimal operating temperature may also be reduced. Secondly, the doping of zinc atoms into indium oxide leads to a lower Fermi energy level moving towards the valence band [27]. This results in an increasing chemical potential gradient, making the reaction with reducing gases more likely to occur and improving responsivity [28]. Thirdly, the distribution of oxygen vacancies is also an influencing factor. When the grain size is large enough, the overall nanowire conductivity will not be affected. However, at nanoscale, the impact of surface effects leads to a concentration difference between surface and bulk, making oxygen vacancies the key factor to sensitivity. In the case of nanowires with high annealing temperatures and slow cooling rates, oxygen vacancies tend to migrate to the surface, reducing free energy and improving gas sensing properties.

## 4. Conclusions

High-quality indium oxide nanowires were synthesized through chemical vapor deposition. Zinc-doped indium oxide nanowires were fabricated in a single-step process. We varied the concentrations of zinc-doped indium oxide nanowires by changing substrate positions and demonstrated the mechanisms of nanowire growth. Accurate electrical measurements were obtained by calculation of the electrical resistivity of a single nanowire and elimination of the contact resistance of the microdevice. Single nanowire electrical measurements reveal that zinc doping increased the resistivity of the indium oxide nanowires, as the valence of zinc is smaller than that of indium, resulting in acceptor doping. XPS analysis confirms that oxygen vacancies increased after doping due to the smaller atomic radius of zinc compared with indium; also, the slow growth process and furnace cooling increased surface oxygen vacancies. The indium oxide nanowires were tested for their response to three reducing gases, acetone, carbon monoxide and ethanol, at temperatures from 150 to 300 °C, with the best response being observed for ethanol at 300 °C. The response of zinc-doped indium oxide nanowires to ethanol at 300 °C reached 40.7, followed by acetone with a response of 13.9, which was better than in previous related studies. The excellent gas sensing performance is attributed to increased oxygen vacancies after doping and the increased adsorption of gas molecules.

## Figures and Tables

**Table 1:** Electrical resistivity measurements for single In_2_O_3_, 3 at% Zn-In_2_O_3_, and 5 at% Zn-In_2_O_3_ nanowire.

	In_2_O_3_ Nanowire	3 at% Zn-In_2_O_3_ Nanowire	5 at% Zn-In_2_O_3_ Nanowire
Resistivity	1.07 × 10^−4^ Ω·cm	2.67 × 10^−4^ Ω·cm	9.31 × 10^−4^ Ω·cm

## Supplementary Materials

The following supporting information can be downloaded at https://www.mdpi.com/article/10.3390/nano13071170/s1, Figure S1: Schematic illustration of the growth mechanism of Zn-In_2_O_3_ nanowires. The reaction followed the VLS route (**a**~**c**).; Figure S2: SEM image of zinc clusters on indium oxide nanowires.; Figure S3: Composition analysis of (**a**) In_2_O_3_ nanowire, (**b**) 3 at% Zn-In_2_O_3_ nanowire, (**c**) 5 at% Zn-In_2_O_3_ nanowire.; Figure S4: The mapping images of (**a**) In_2_O_3_ nanowire (**b**) 3 at% Zn-In_2_O_3_ nanowire (**c**) 5 at% Zn-In_2_O_3_ nanowire.; Figure S5: (**a**,**b**) SEM images of single In_2_O_3_ nanowire-based electrical measurement micro-device. I-V measurements of single In_2_O_3_ nanowire. (**c**) R_13+_ (**d**) R_13-_ (**e**) R_14+_ (**f**) R_14-_ (**g**) R_23+_ (**h**) R_23-_ (**i**) R_24+_ (**j**) R_24-_; Figure S6: (**a**,**b**) SEM image of single 3 at% Zn-In_2_O_3_ nanowire-based electrical measurement micro-device. I-V measurements of 3 at% Zn- In_2_O_3_ nanowire. (**c**) R_13+_ (**d**) R_13-_ (**e**) R_14+_ (**f**) R_14-_ (**g**) R_23+_ (**h**) R_23-_ (**i**) R_24+_ (**j**) R_24-_.; Figure S7: (**a**,**b**) SEM image of single 5 at% Zn-In_2_O_3_ nanowire-based electrical measurement micro-device. I-V measurements of 5 at% Zn-In_2_O_3_ nanowire. (**c**) R_13+_ (**d**) R_13-_ (**e**) R_14+_ (**f**) R_14-_ (**g**) R_23+_ (**h**) R_23-_ (**i**) R_24+_ (**j**) R_24-_.; Figure S8: (**a**–**d**) Dynamic gas sensing curves of single In_2_O_3_ nanowire, 3 at% Zn-In_2_O_3_ nanowire and 5 at% Zn-In_2_O_3_ nanowire at 150 °C, 200 °C, 250 °C and 300 °C to 5 ppm acetone.; Figure S9: (**a**–**d**) Dynamic gas sensing curves of single In_2_O_3_ nanowire, 3 at% Zn-In_2_O_3_ nanowire and 5 at% Zn-In_2_O_3_ nanowire at 150 °C, 200 °C, 250 °C, and 300 °C to 5 ppm carbon monoxide.; Figure S10: (**a**–**d**) Dynamic gas sensing curves of single In_2_O_3_ nanowire, 3 at% Zn-In_2_O_3_ nanowire and 5 at% Zn-In_2_O_3_ nanowire at 150 °C, 200 °C, 250 °C and 300 °C to 5 ppm ethanol.; Figure S11: Schematic illustration of the change of electron depletion region after n-type semiconductor absorbs reducing gas.; Table S1: Summary of gas sensing performance of In_2_O_3_, 3 at% Zn-In_2_O_3_, 5 at% Zn-In_2_O_3_ nanowires.; Table S2: Comparison with previous sensing studies on In_2_O_3_ nanowires with different dopants for 5 ppm ethanol.; Table S3: Comparison with previous sensing studies on In_2_O_3_ nanostructures with different dopants for 5 ppm acetone.
