# Supplementary material for: Controlled Synthesis and Enhanced Gas Sensing Performance of Zinc-Doped Indium Oxide Nanowires
Source: Nanomaterials (Basel). 2023 Mar 25;13(7):1170. doi: 10.3390/nano13071170 (PMC10097380; doi:10.3390/nano13071170)
Supplement: Supplementary file 1 [file nanomaterials-13-01170-s001.zip › nanomaterials-2283270-supplementary-conversion.pdf]

Supporting Information

Controlled Synthesis and Enhanced Gas Sensing Performance of Zinc-Doped Indium Oxide Nanowires

Che-Wen Yu [1], Hsuan-Wei Fu [1], Shu-Meng Yang [1], Yu-Shan Lin [1] and Kuo-Chang Lu [1,2*]

[1] Department of Materials Science and Engineering, National Cheng Kung University, Tainan 701, Taiwan; n56091352@gs.ncku.edu.tw (C.-W.Y.); n56091556@gs.ncku.edu.tw (H.-W.F.); n56074287@gs.ncku.edu.tw (S.-M.Y.); n56114053@gs.ncku.edu.tw (Y.-S.L.)

[2] Core Facility Center, National Cheng Kung University, Tainan 701, Taiwan

* Correspondence: gkclu@mail.ncku.edu.tw; Tel.: +886-6-275-7575 (ext. 62920)

List of contents

Figure S1 | Schematic illustration of the growth mechanism of Zn-In_2O_3 nanowires. The reaction followed the VLS route(a)~(c).

Figure S2 | SEM image of zinc clusters on indium oxide nanowires.

Figure S3 | Composition analysis of(a) In_2O_3 nanowire (b) 3 at% Zn-In_2O_3 nanowire (c) 5 at% Zn-In_2O_3 nanowire.

Figure S4 | The mapping images of(a) In_2O_3 nanowire (b) 3 at% Zn-In_2O_3 nanowire (c) 5 at% Zn-In_2O_3 nanowire.

Figure S5 | (a,b) SEM images of single In_2O_3 nanowire-based electrical measurement micro-device. I-V measurements of single In_2O_3 nanowire. (c) R_{13+} (d) R_{13-} (e) R_{14+} (f) R_{14-} (g) R_{23+} (h) R_{23-} (i) R_{24+} (j) R_{24-}.

Figure S6 | (a,b) SEM image of single 3 at% Zn-In_2O_3 nanowire-based electrical measurement micro-device. I-V measurements of 3 at% Zn- In_2O_3 nanowire. (c) R_{13+} (d) R_{13-} (e) R_{14+} (f) R_{14-} (g) R_{23+} (h) R_{23-} (i) R_{24+} (j) R_{24-}.

Figure S7 | (a,b) SEM image of single 5 at% Zn-In_2O_3 nanowire-based electrical measurement micro-device. I-V measurements of 5 at% Zn-In_2O_3 nanowire. (c) R_{13+} (d) R_{13-} (e) R_{14+} (f) R_{14-} (g) R_{23+} (h) R_{23-} (i) R_{24+} (j) R_{24-}.

Figure S8 | (a-d) Dynamic gas sensing curves of single In_2O_3 nanowire, 3 at% Zn-In_2O_3 nanowire and 5 at% Zn-In_2O_3 nanowire at 150 °C, 200 °C, 250 °C and 300 °C to 5 ppm acetone.

Figure S9 | (a-d) Dynamic gas sensing curves of single In_2O_3 nanowire, 3 at% Zn-In_2O_3 nanowire and 5 at% Zn-In_2O_3 nanowire at 150 °C, 200 °C, 250 °C, and 300 °C to 5 ppm carbon monoxide.

Figure S10 | (a-d) Dynamic gas sensing curves of single In_2O_3 nanowire, 3 at% Zn-In_2O_3 nanowire and 5 at% Zn-In_2O_3 nanowire at 150 °C, 200 °C, 250 °C and 300 °C to 5 ppm ethanol.

Figure S11 | Schematic illustration of the change of electron depletion region after n-type semiconductor absorbs reducing gas.

Table S1 | Summary of gas sensing performance of In_2O_3, 3 at% Zn-In_2O_3, 5 at% Zn-In_2O_3 nanowires.

Table S2 | Comparison with previous sensing studies on In_2O_3 nanowires with different dopants for

5ppm ethanol.

Table S3 | Comparison with previous sensing studies on In_2O_3 nanostructures with different dopants for 5ppm acetone.

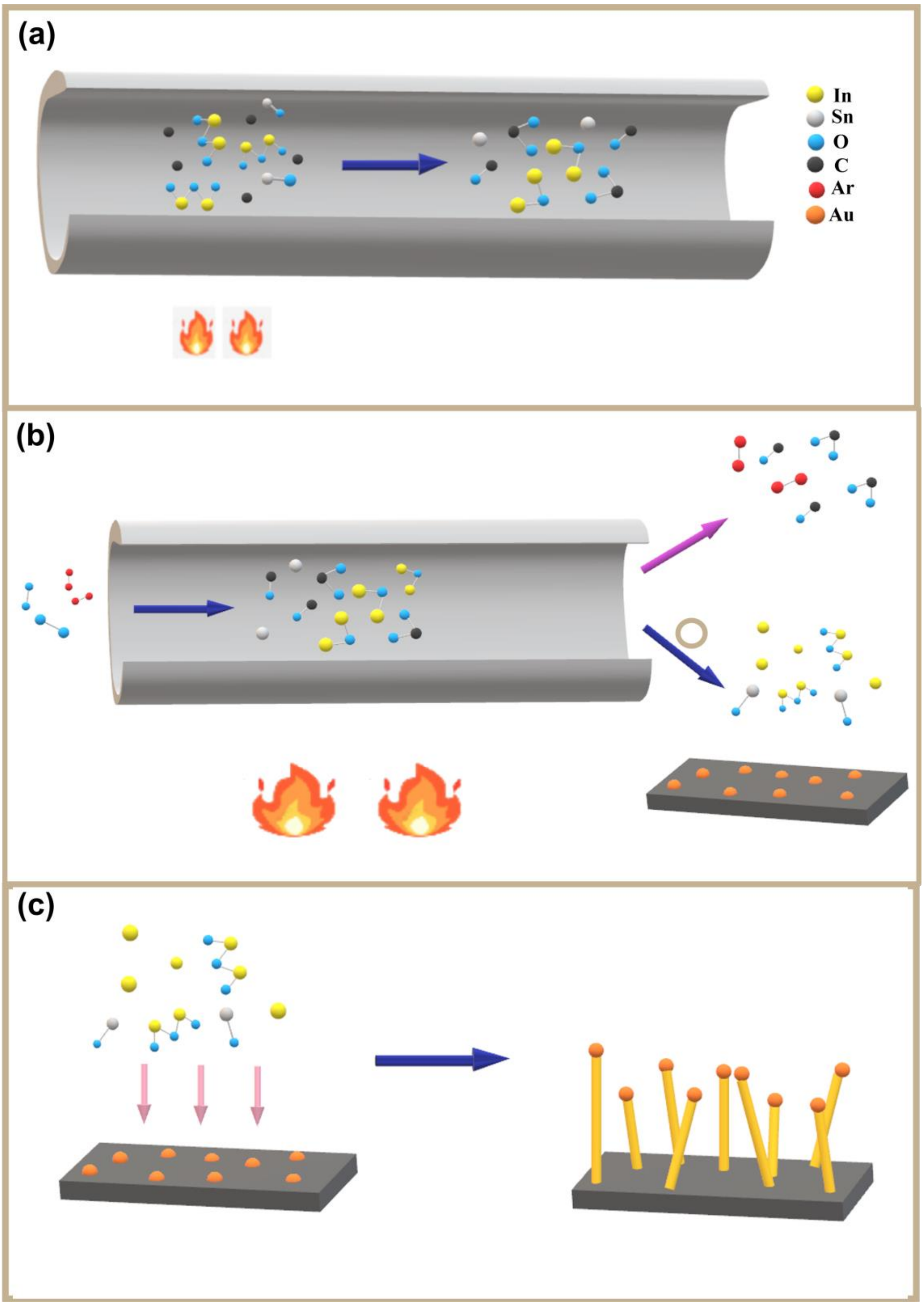

Figure S1 Schematic illustration of the growth mechanism of Zn-In_2O_3 nanowires. The reaction followed the VLS route(a)~(c).

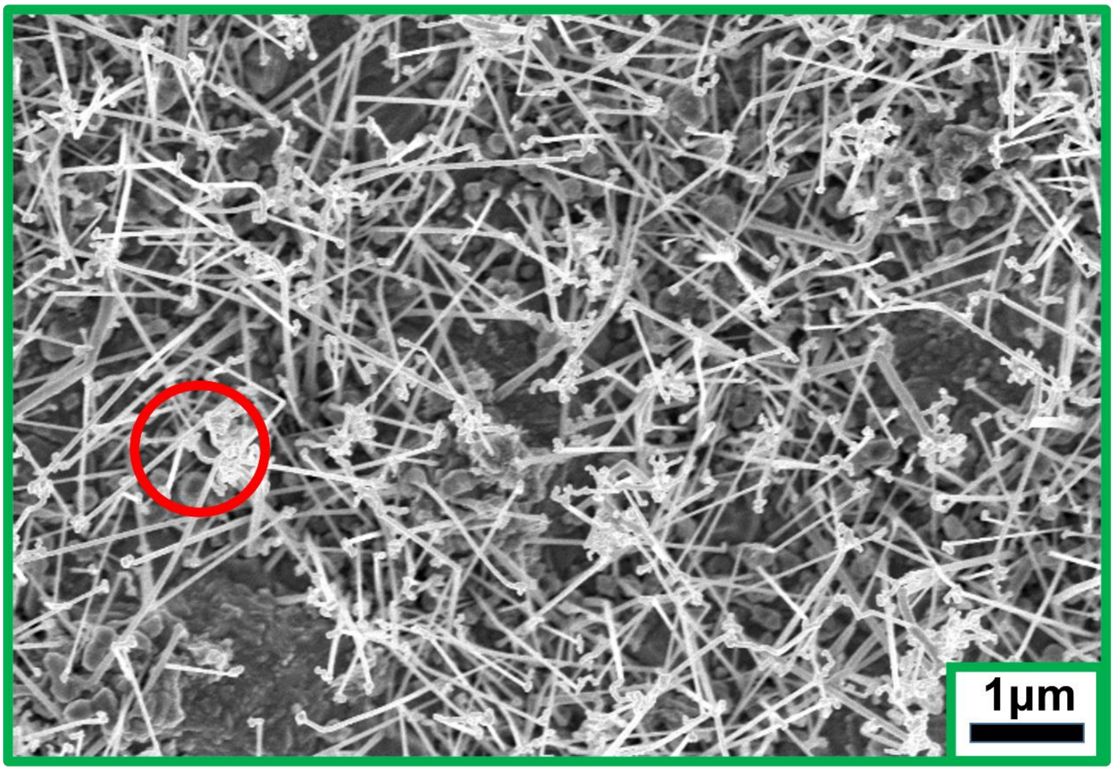

Figure S2 SEM image of zinc clusters on indium oxide nanowires.

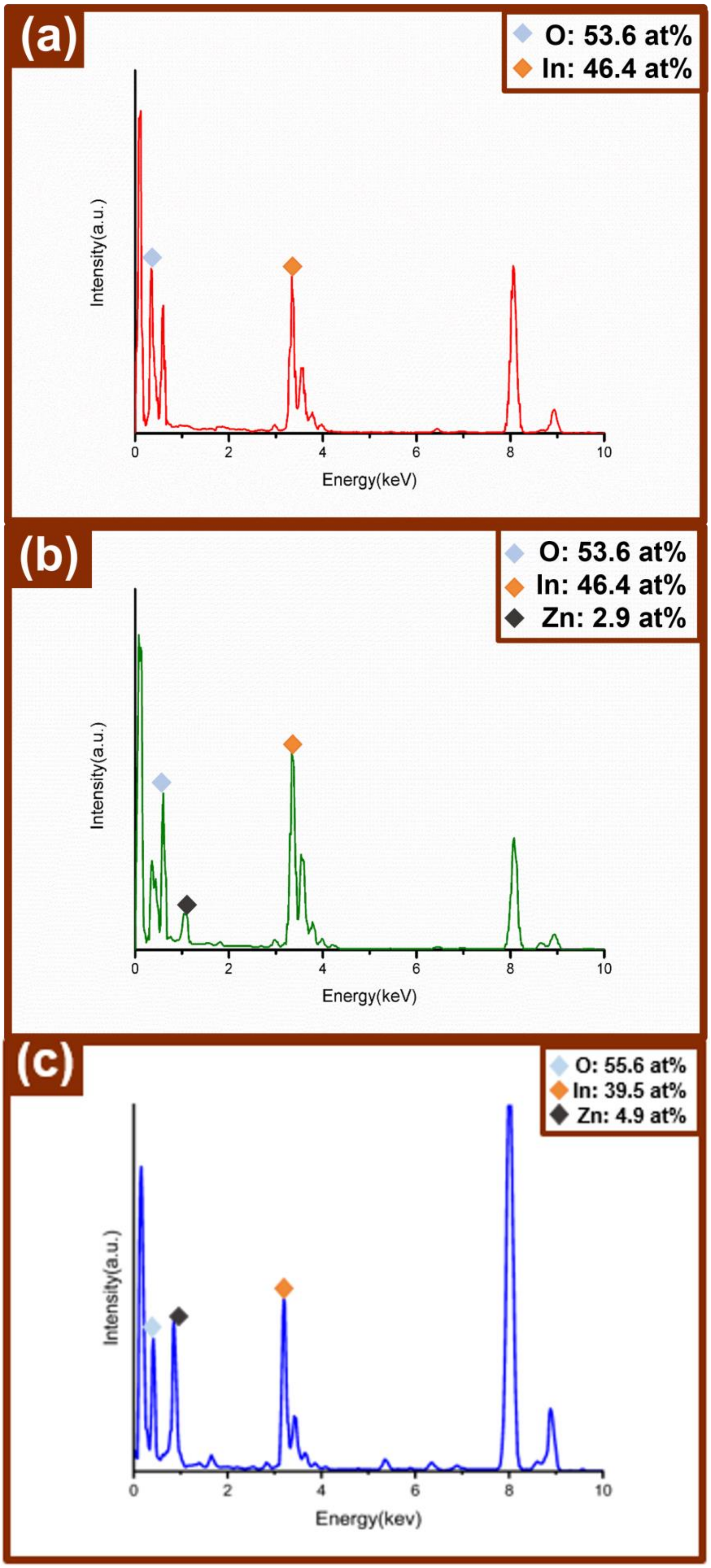

(a)
O: 53.6 at%
In: 46.4 at%
Intensity(a.u.)
Energy(keV)
0
2
4
6
8
10
(b)
O: 53.6 at%
In: 46.4 at%
Zn: 2.9 at%
Intensity(a.u.)
Energy(keV)
0
2
4
6
8
10
(c)
O: 55.6 at%
In: 39.5 at%
Zn: 4.9 at%
Intensity(a.u.)
Energy(kev)
0
2
4
6
8
10

Figure S3 Composition analysis of(a) In_2O_3 nanowire (b) 3 at% Zn-In_2O_3 nanowire (c) 5 at% Zn-In_2O_3 nanowire.

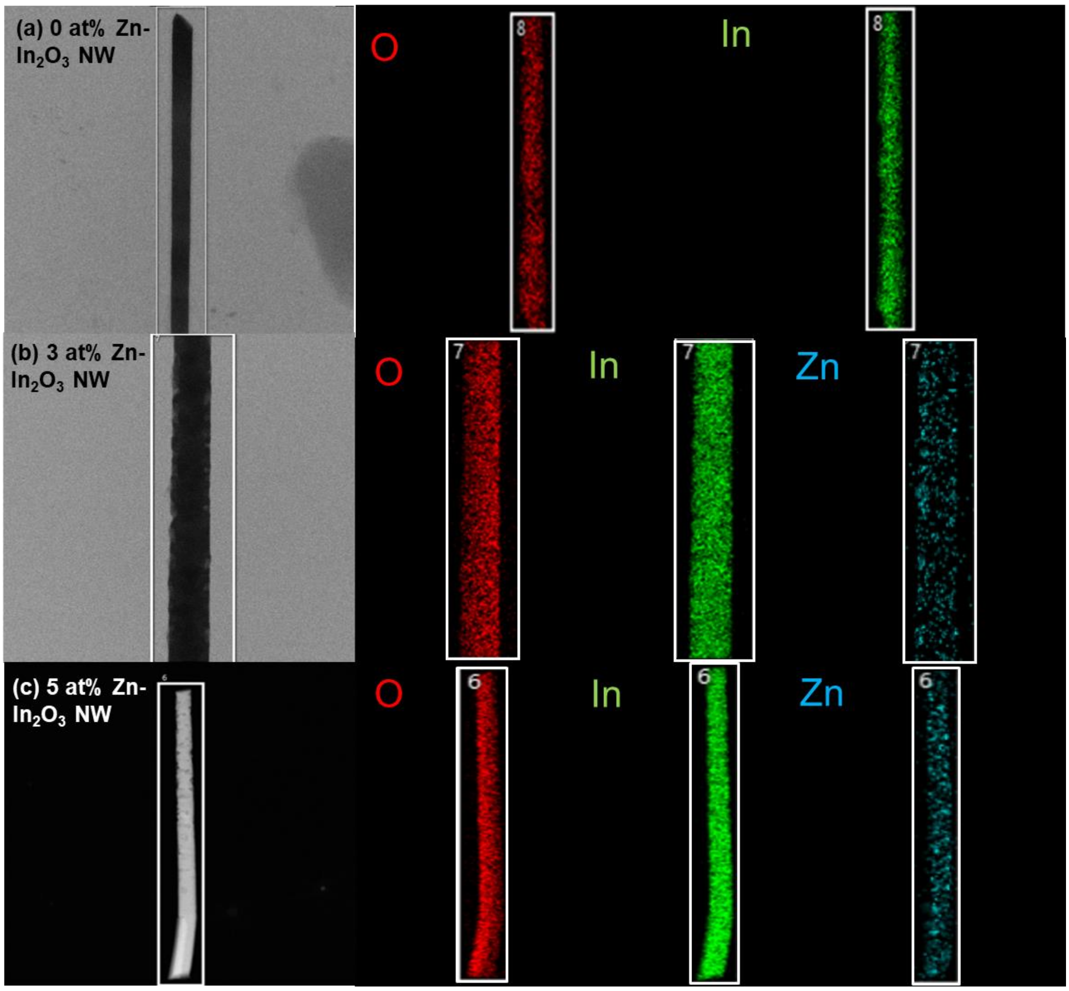

Figure S4 The mapping images of(a) In_2O_3 nanowire (b) 3 at% Zn-In_2O_3 nanowire (c) 5 at% Zn-In_2O_3 nanowire.

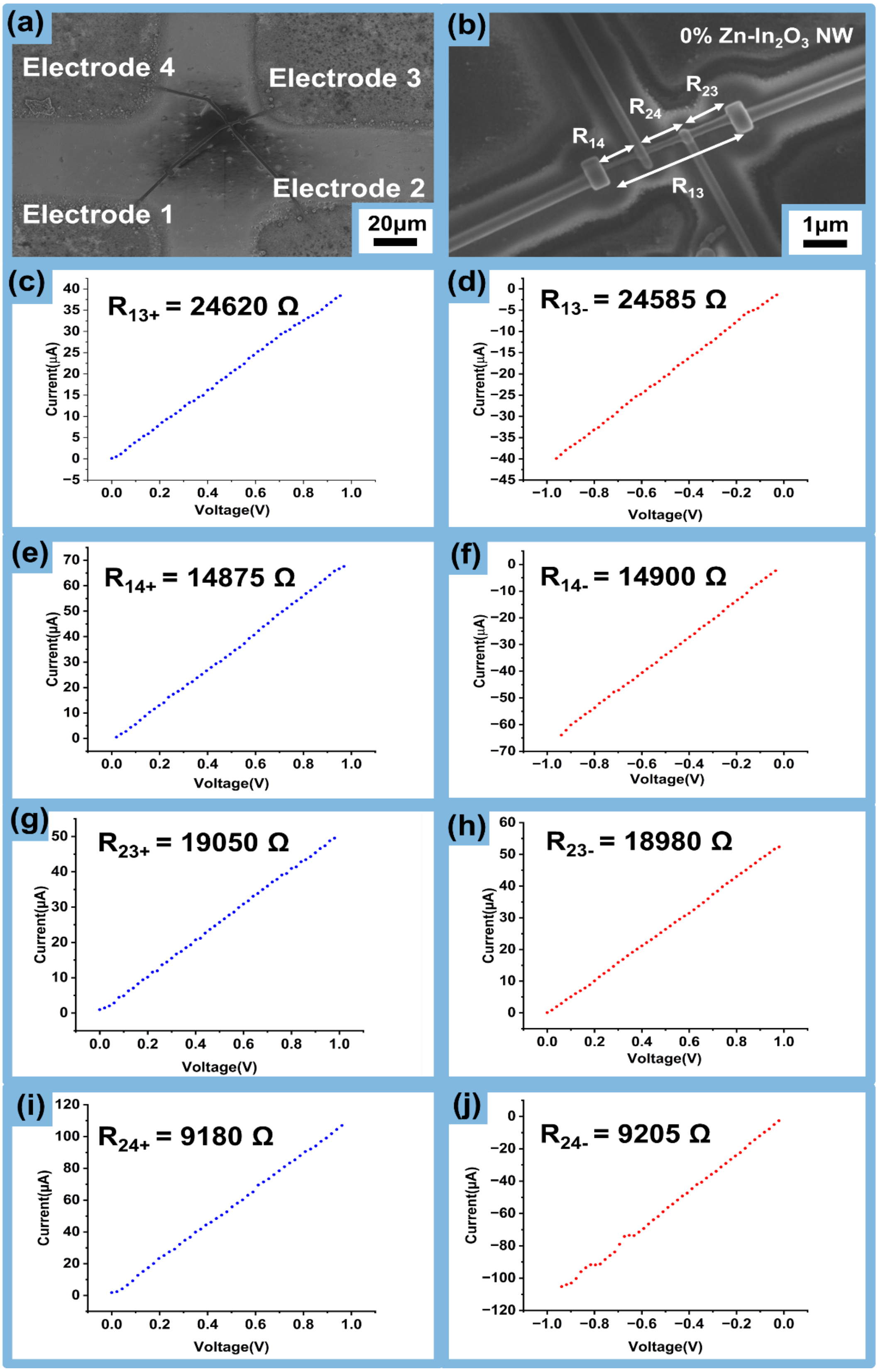

Figure S5. (a,b) SEM images of single In_2O_3 nanowire-based electrical measurement micro-device. I-V measurements of single In_2O_3 nanowire. (c) R_{13+} (d) R_{13-} (e) R_{14+} (f) R_{14-} (g) R_{23+} (h) R_{23-} (i) R_{24+} (j) R_{24-}.

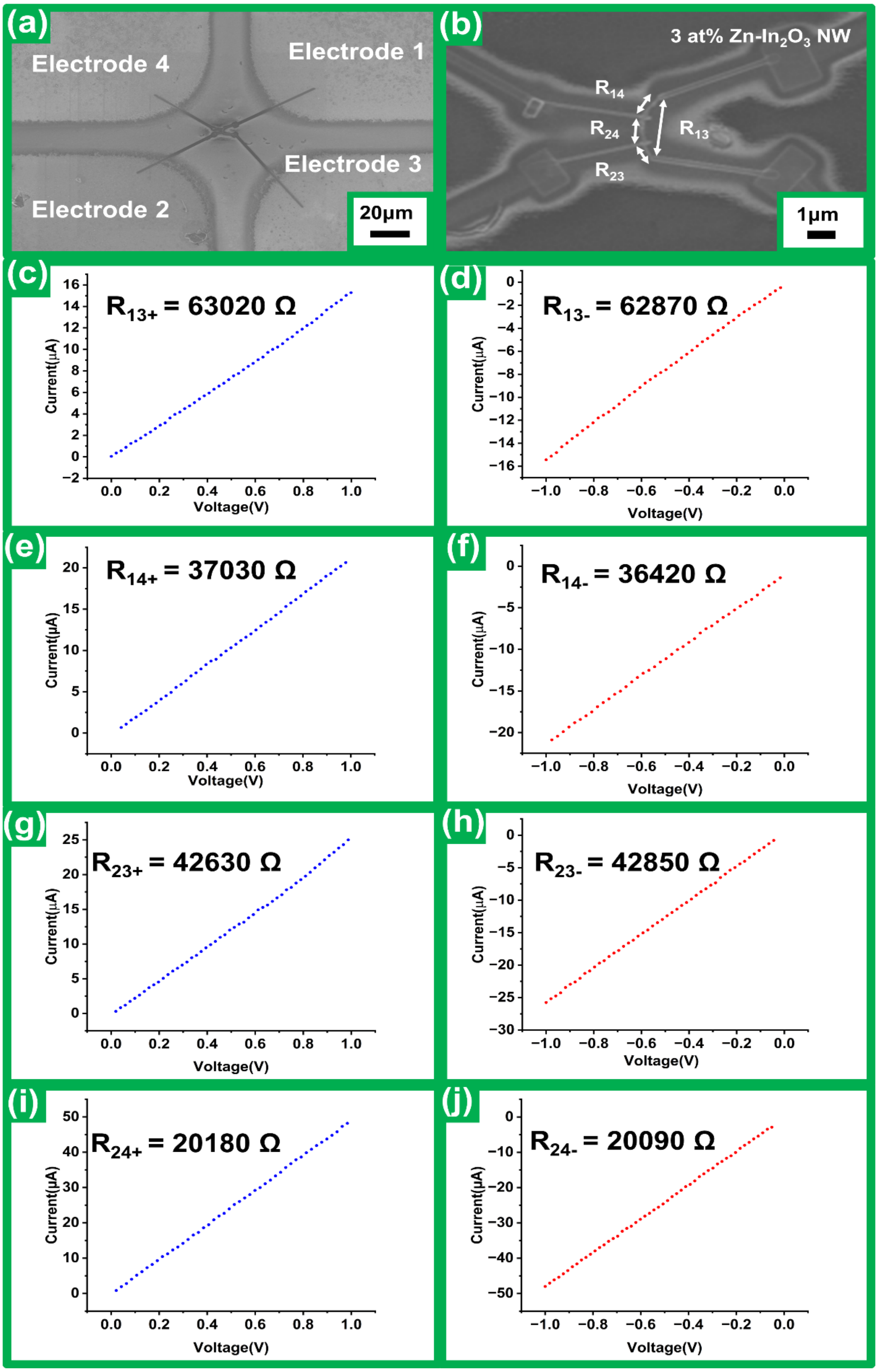

Figure S6. (a,b) SEM image of single 3 at% Zn-In_2O_3 nanowire-based electrical measurement micro-device. I-V measurements of 3 at% Zn- In_2O_3 nanowire. (c) R_{13+} (d) R_{13-} (e) R_{14+} (f) R_{14-} (g) R_{23+} (h) R_{23-} (i) R_{24+} (j) R_{24-}.

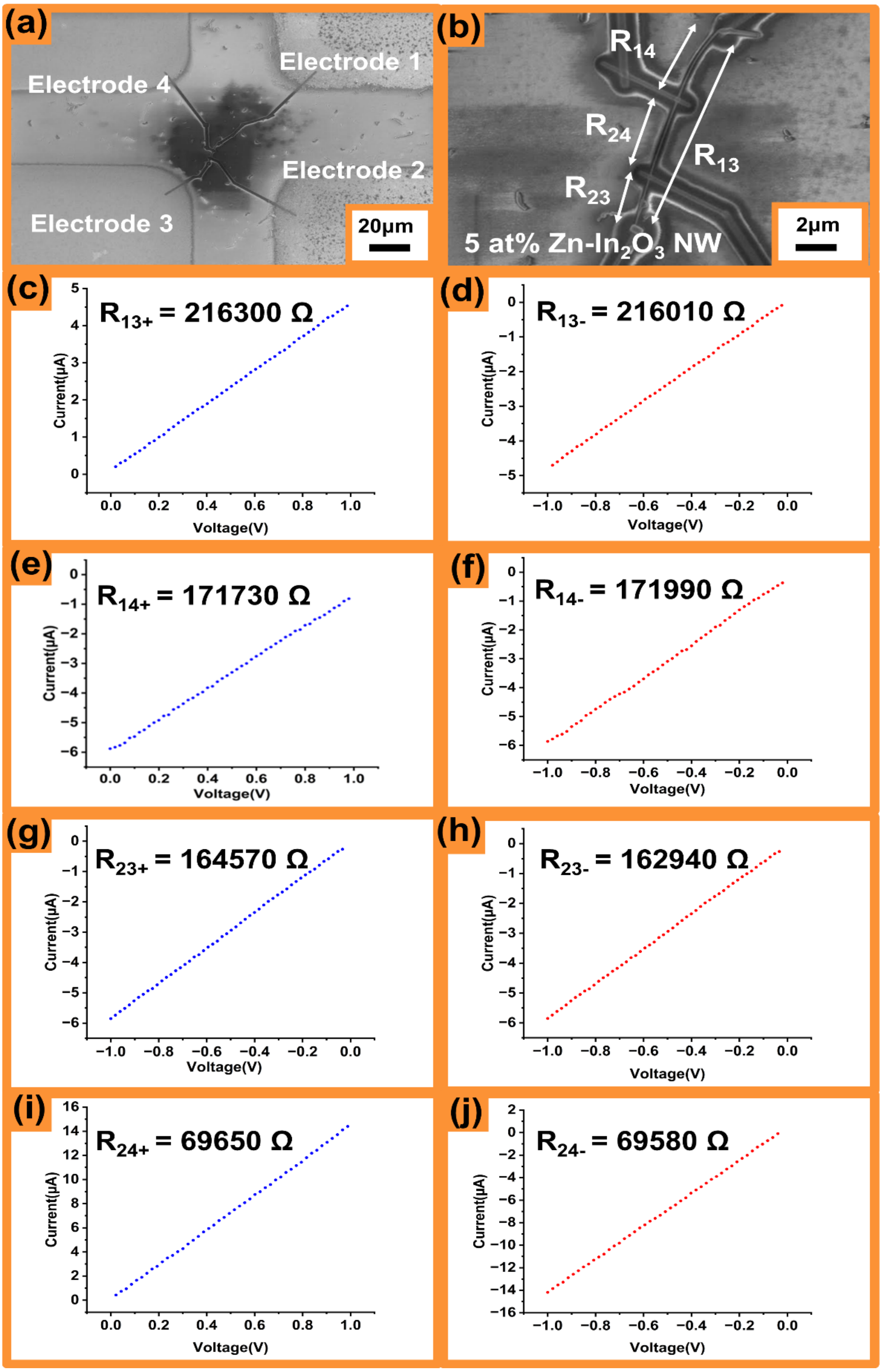

Figure S7. (a,b) SEM image of single 5 at% Zn-In_2O_3 nanowire-based electrical measurement micro-device. I-V measurements of 5 at% Zn-In_2O_3 nanowire. (c) R_{13+} (d) R_{13-} (e) R_{14+} (f) R_{14-} (g) R_{23+} (h) R_{23-} (i) R_{24+} (j) R_{24-}.

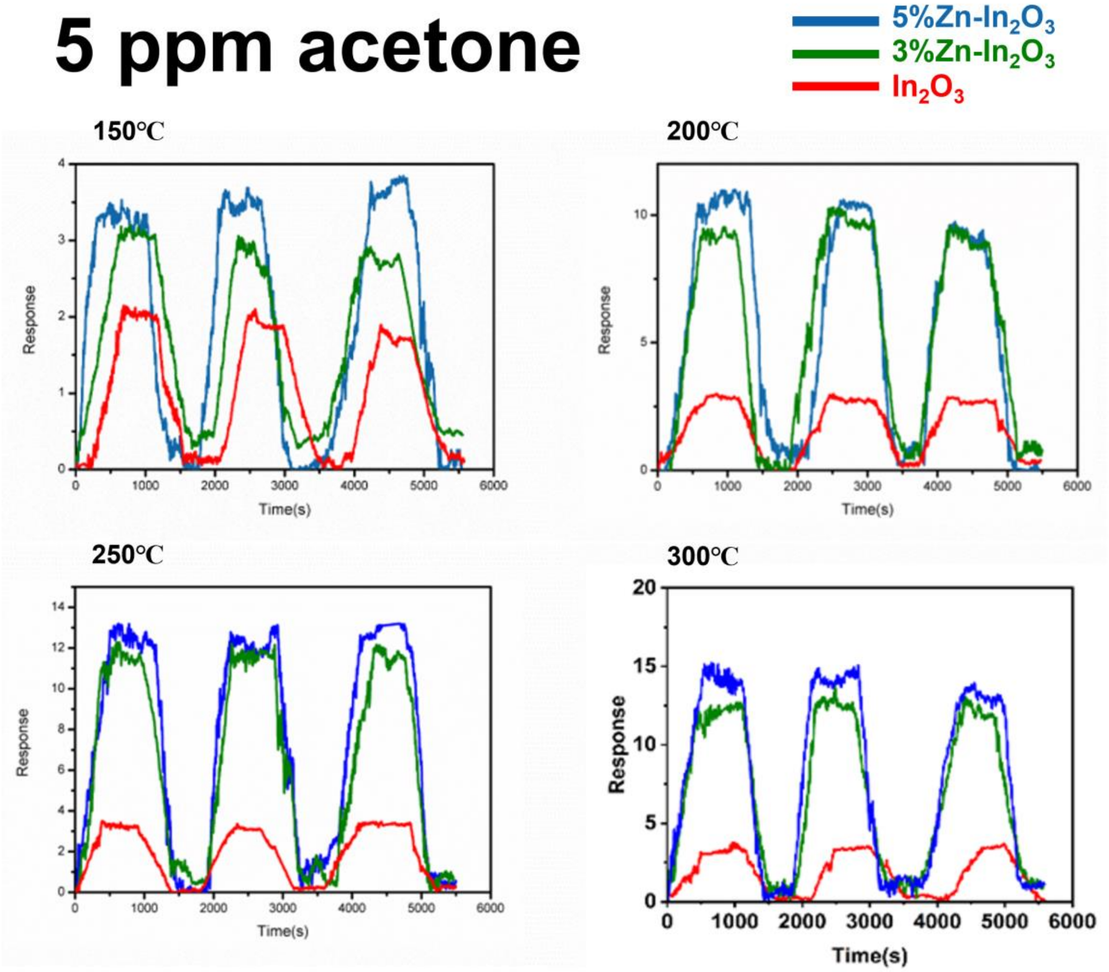

Figure S8. (a-d) Dynamic gas sensing curves of single In_2O_3 nanowire, 3 at% Zn-In_2O_3 nanowire and 5 at% Zn-In_2O_3 nanowire at 150 °C, 200 °C, 250 °C and 300 °C to 5 ppm acetone.

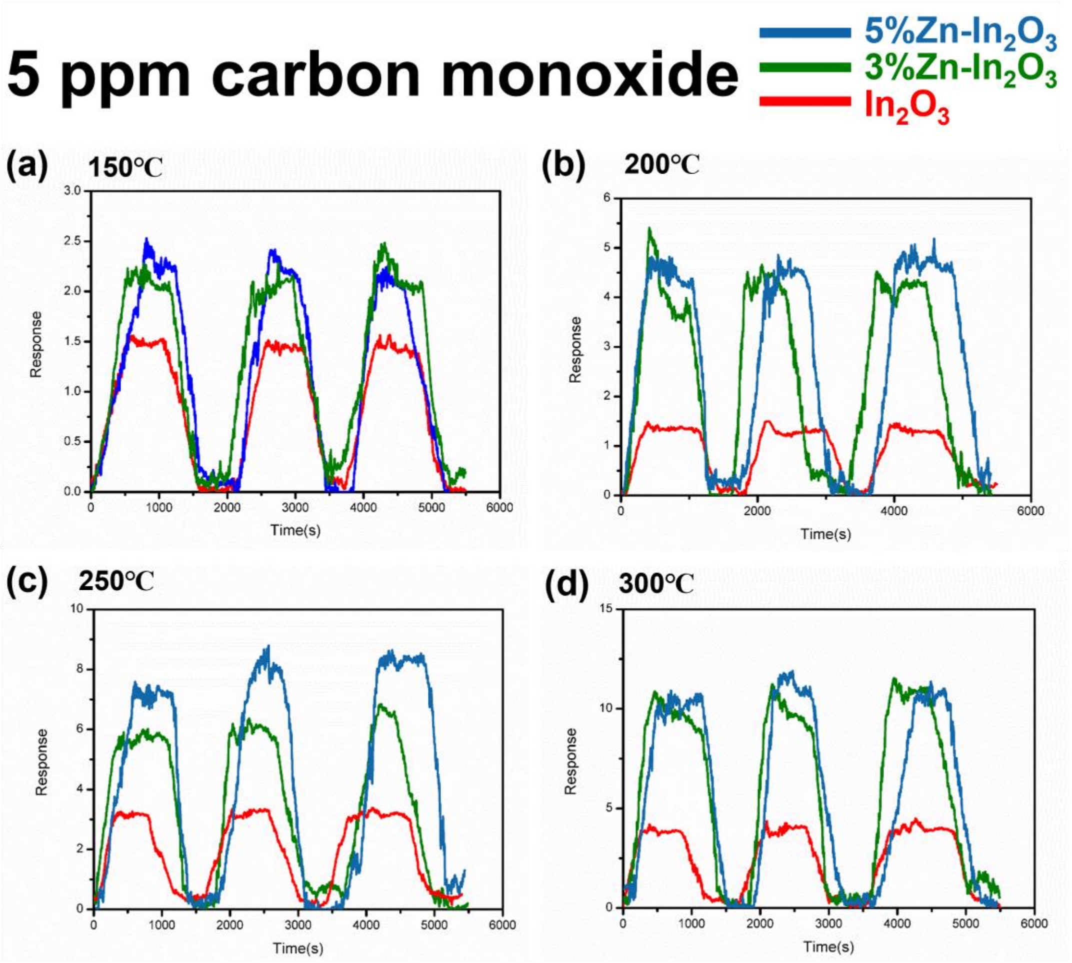

Figure S9. (a-d) Dynamic gas sensing curves of single In_2O_3 nanowire, 3 at% Zn-In_2O_3 nanowire and 5 at% Zn-In_2O_3 nanowire at 150 °C, 200 °C, 250 °C, and 300 °C to 5 ppm carbon monoxide.

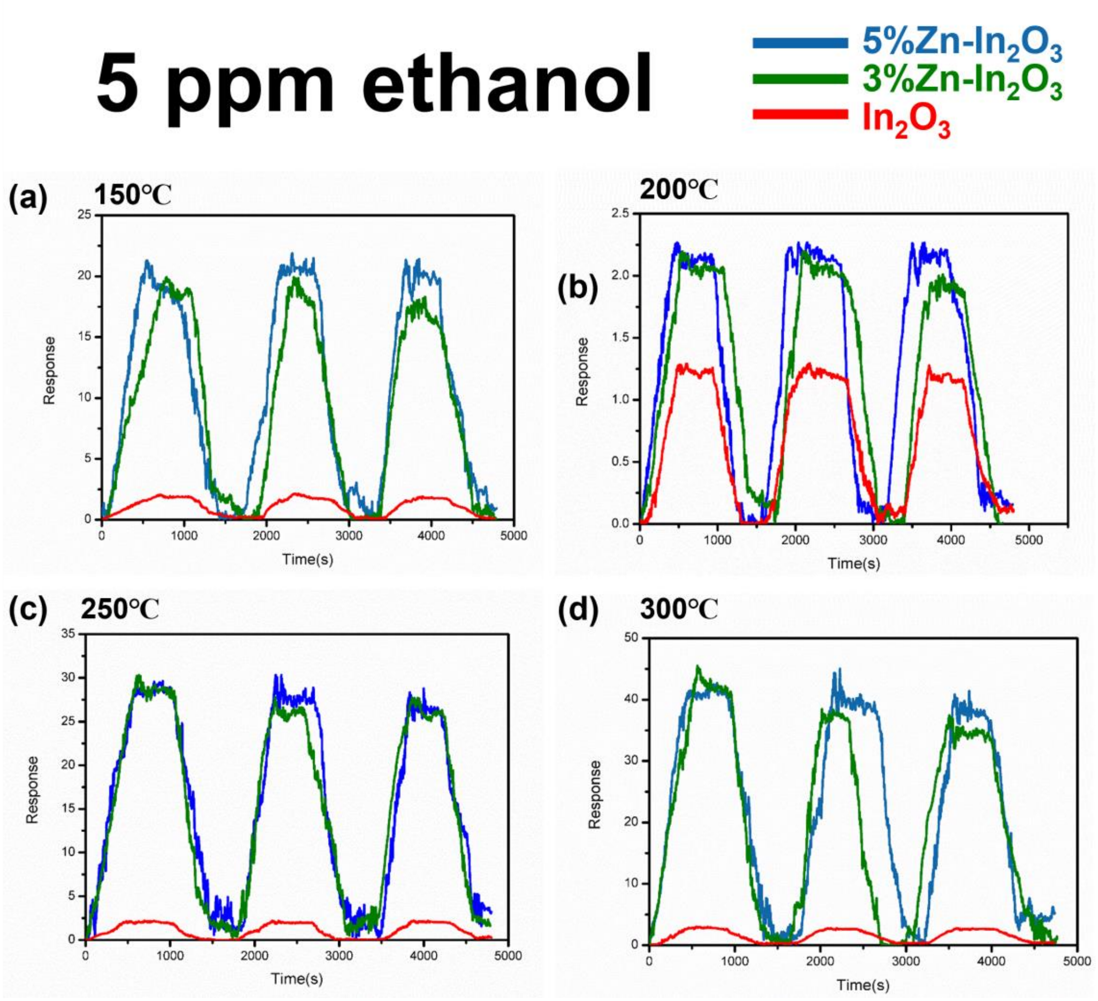

Figure S10. (a-d) Dynamic gas sensing curves of single In_2O_3 nanowire, 3 at% Zn-In_2O_3 nanowire and 5 at% Zn-In_2O_3 nanowire at 150 °C, 200 °C, 250 °C and 300 °C to 5 ppm ethanol.

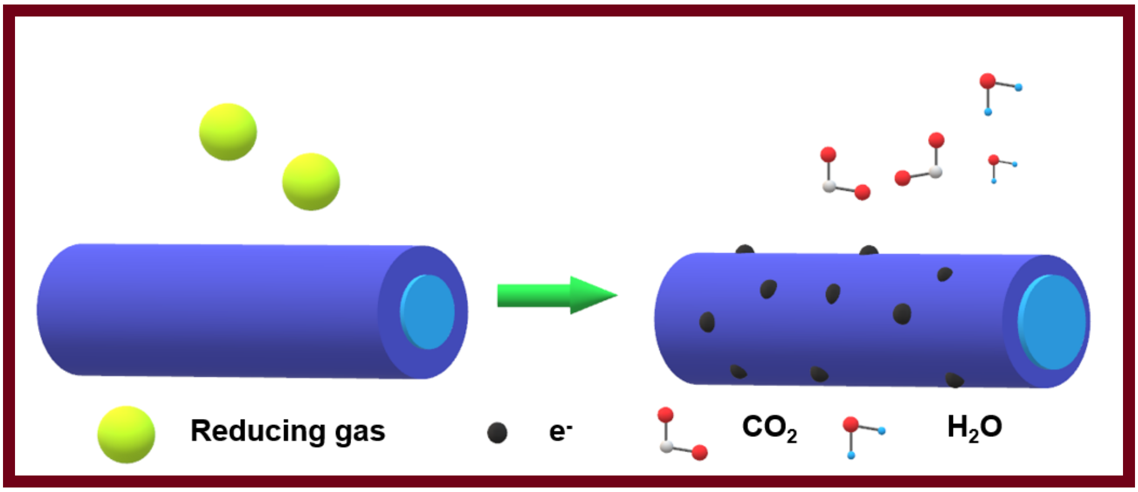

Figure S11. Schematic illustration of the change of electron depletion region after n-type semiconductor absorbs reducing gas.

Table S1. Summary of gas sensing performance of In_2O_3, 3 at% Zn-In_2O_3, 5 at% Zn-In_2O_3 nanowires.

Acetone	150°C	200°C	250°C	300°C
In_2O_3	2.0	2.9	3.5	3.4
3% Zn-In_2O_3	2.8	9.9	12.1	12.3
5% Zn-In_2O_3	3.5	10.9	13.2	13.9
Carbon monoxide	150°C	200°C	250°C	300°C
In_2O_3	1.5	1.2	3.2	3.8
3% Zn-In_2O_3	2.2	3.8	6.1	10.0
5% Zn-In_2O_3	2.3	4.5	7.2	10.8
Ethanol	150°C	200°C	250°C	300°C
In_2O_3	1.3	1.8	2.2	2.5
3% Zn-In_2O_3	2.2	18.1	30.7	36.8
5% Zn-In_2O_3	2.3	20.1	29.7	40.7

Table S2. Comparison with previous sensing studies on In_2O_3 nanowires with different dopants for 5ppm ethanol.

Sensing element	Gas concentration	Temperature	Response	Reference
In_2O_3 NW	5ppm	300°C	2.5	This work
3% Zn-doped In_2O_3 NW	5ppm	300°C	36.8	This work
5% Zn-doped In_2O_3 NW	5ppm	300°C	40.7	This work
In_2O_3 NWs	20ppm	300°C	30	[5]
5% Sn-doped In2O3 NWs	10ppm	400°C	5	[22]
0.5% Co-doped In2O3 NWs	100ppm	300°C	16	[23]

Table S3. Comparison with previous sensing studies on In_2O_3 nanostructures with different dopants for 5ppm acetone.

Sensing element	Gas concentration	Temperature	Response	Reference
In_2O_3 NW	5ppm	300°C	3.4	This work
3% Zn-doped In_2O_3 NW	5ppm	300°C	12.3	This work
5% Zn-doped In_2O_3 NW	5ppm	300°C	13.9	This work
Electrospinning In_2O_3 NWs	10ppm	200°C	10	[24]
3% Ag-doped In2O3 NPs	50ppm	300°C	7	[25]
0.5% Co-doped In2O3 NPs	5ppm	240°C	4	[26]
